# The PII signaling protein from red algae represents an evolutionary link between cyanobacterial and Chloroplastida PII proteins

**DOI:** 10.1038/s41598-017-19046-7

**Published:** 2018-01-15

**Authors:** Tatyana Lapina, Khaled A. Selim, Karl Forchhammer, Elena Ermilova

**Affiliations:** 1 Biological Faculty, Saint-Petersburg State University, Universitetskaya nab. 7/9, Saint-Petersburg, 199034 Russia; 2 Interfaculty Institute of Microbiology and Infection Medicine/Organismic Interactions Department, Eberhard-Karls-Universität Tübingen, Auf der Morgenstelle 28, 72076 Tübingen, Germany

## Abstract

PII superfamily consists of widespread signal transduction proteins found in all domains of life. Whereas they are well-studied in Archaea, Bacteria and Chloroplastida, no PII homolog has been analyzed in Rhodophyta (red algae), where PII is encoded by a chloroplast localized *glnB* gene. Here, we characterized relevant sensory properties of PII from the red alga *Porphyra purpurea* (PpPII) in comparison to PII proteins from different phyla of oxygenic phototrophs (cyanobacteria, *Chlamydomonas* and *Physcomitrella*) to assess evolutionary conservation versus adaptive properties. Like its cyanobacterial counterparts, PpPII binds ATP/ADP and 2-oxoglutarate in synergy with ATP. However, green algae and land plant PII proteins lost the ability to bind ADP. In contrast to PII proteins from green algae and land plants, PpPII enhances the activity of N-acetyl-L-glutamate kinase (NAGK) and relieves it from feedback inhibition by arginine in a glutamine-independent manner. Like PII from Chloroplastida, PpPII is not able to interact with the cyanobacterial transcriptional co-activator PipX. These data emphasize the conserved role of NAGK as a major PII-interactor throughout the evolution of oxygenic phototrophs, and confirms the specific role of PipX for cyanobacteria. Our results highlight the PII signaling system in red algae as an evolutionary intermediate between Cyanobacteria and Chlorophyta.

## Introduction

The PII superfamily were originally described as widely distributed members of a family of cell signaling proteins occurring in all domains of life^1–3^ with representatives in almost all bacteria and in nitrogen-fixing archaea^4,5^ as well as in oxygenic eukaryotic phototrophs^6^. The canonical PII proteins are the master regulator of nitrogen metabolism and they are encoded by *glnB* and *glnK* genes^2,7^. The superfamily of PII-like proteins was enlarged by including members that are characterized by the typical structural architecture of PII proteins but lack the typical PROSITE signature pattern of initially characterized PII proteins^7^. Those PII homologues, which contain the typically conserved PROSITE motifs of GlnB/GlnK-like PII proteins are referred as canonical PII proteins.

The canonical PII proteins have fundamentals roles as energy/carbon/nitrogen sensors^8^. The binding of small effector molecules to PII proteins allows modulation of different cellular functions. Competitive binding of ATP or ADP and synergistic binding of 2-oxoglutarate (2-OG) with ATP enables PII to estimate the current energy and nitrogen/carbon status of the cells. The various effector molecule binding events cause signaling through conformational changes within the PII tirmer, which in turn allows PII to bind to different interacting partners to regulate the actual metabolic situation. Under conditions of high 2-OG levels (poor nitrogen supply), the ATP-dependent binding of 2-OG to PII causes strong conformational changes in the T-loop, which in turn impairs the interaction of PII proteins with different targets^7^. In all examined cases studied so far, PII proteins coordinate the central C/N anabolic metabolism by regulating the activity of transcription factors, key metabolic enzymes and transporters^9–12^.

In many bacteria, PII proteins of the GlnB subfamily are engaged in glutamine synthetase control at various levels^13,14^ whereas PII proteins of the GlnK subfamily regulate ammonia transporter Amt by reversibly clogging its NH_3_ channel^11^. In cyanobacteria, PII proteins are present in all known species. In contrast to PII proteins from other bacterial lineages, cyanobacterial PII proteins have evolved to regulate N-acetyl-L-glutamate kinase (EC 2.7.2.8) (NAGK), which is the controlling enzyme of arginine biosynthesis^15^, and the general nitrogen control transcription factor NtcA through binding to the transcriptional co-activator PipX^16–18^. In common with other bacterial PII proteins, cyanobacterial PII proteins interact with the BCCP subunit of the acetyl-CoA carboxylase enzyme complex^19^ to control cellular acetyl-CoA levels.

Eukaryotic homologues of canonical PII proteins are restricted to members of the plant kingdom. In Chloroplastida (green algae and land plants) they are nuclear-encoded and, in Rhodophyta they are coded by the plastid genome^6^. Genomic information from red algae revealed that PII signaling has been lost in some families of the red algae^3,6^ whereas it is present in three members of Bangiophyceae family, *Porphyra purpurea, Porphyra umbilicalis* and *Pyropia yezoensis*^20,21^. It is of note that the chloroplasts of red algae have more archaic properties than those of the Chloroplastida and are probably more similar to the cyanobacterial endosymbiont that gave rise to chloroplasts. Many genes that were transferred from the chloroplast to the nucleus during the evolution of Chlorophyta are still encoded in the red algae chloroplast, such as the *glnB* gene (encoding PII) or the *argB* gene (encoding NAGK) and can thus be considered as evolutionary relics. In Chloroplastida, PII and NAGK are localized in the chloroplast^22,23^, where the PII proteins control the ornithine pathway via activity regulation of NAGK, as in cyanobacteria^24^. However, in green algae and land plants, NAGK activity-regulation responds - via a specific feature of the respective PII proteins - to the cellular glutamine levels in addition to the primary effects ATP and 2-OG^24,25^. A short additional C-terminal segment, absent in bacterial PII proteins, was shown to act as low-affinity glutamine binding site. This segment seems to have evolved when the *glnB* gene from the endosymbiont was translocated to the nucleus, since it is absent in Rhodophyta, where this translocation has not taken place. This suggests that the PII signaling protein from red algae resembles the PII signaling system of the early endosymbiont. However, functional analysis of red algal PII proteins is necessary to close the gap in our understanding of PII evolution in oxygenic phototrophs.

*P. purpurea* has been one of the important reference red algae for genomic and biochemical studies of many cellular processes including nitrogen metabolism^20,26–28^. For this reason, we started studying PII signaling in *P. purpurea*, in particular with respect to the sensory binding of small effector molecules, and compared it to the binding properties of PII proteins across different phyla representing the evolution of oxygenic phototrophs, from the cyanobacterium *Synechococcus elongatus* PCC 7942 (SyPII), through green algal *Chlamydomonas reinhardtii* (CrPII) to the moss plant *Physcomitrella patens* (PhyscoPII). Furthermore, we report PpPII-mediated NAGK regulation, with the question in mind, whether it is resembles NAGK control in cyanobacteria or green plants. Special attention was given to examine the possibility that PpPII might still be able to control the cyanobacterial PipX transcription co-activator, which is absent from Chlorophyta.

## Results

### Sequence based alignments of PII proteins

**Figure 1:**
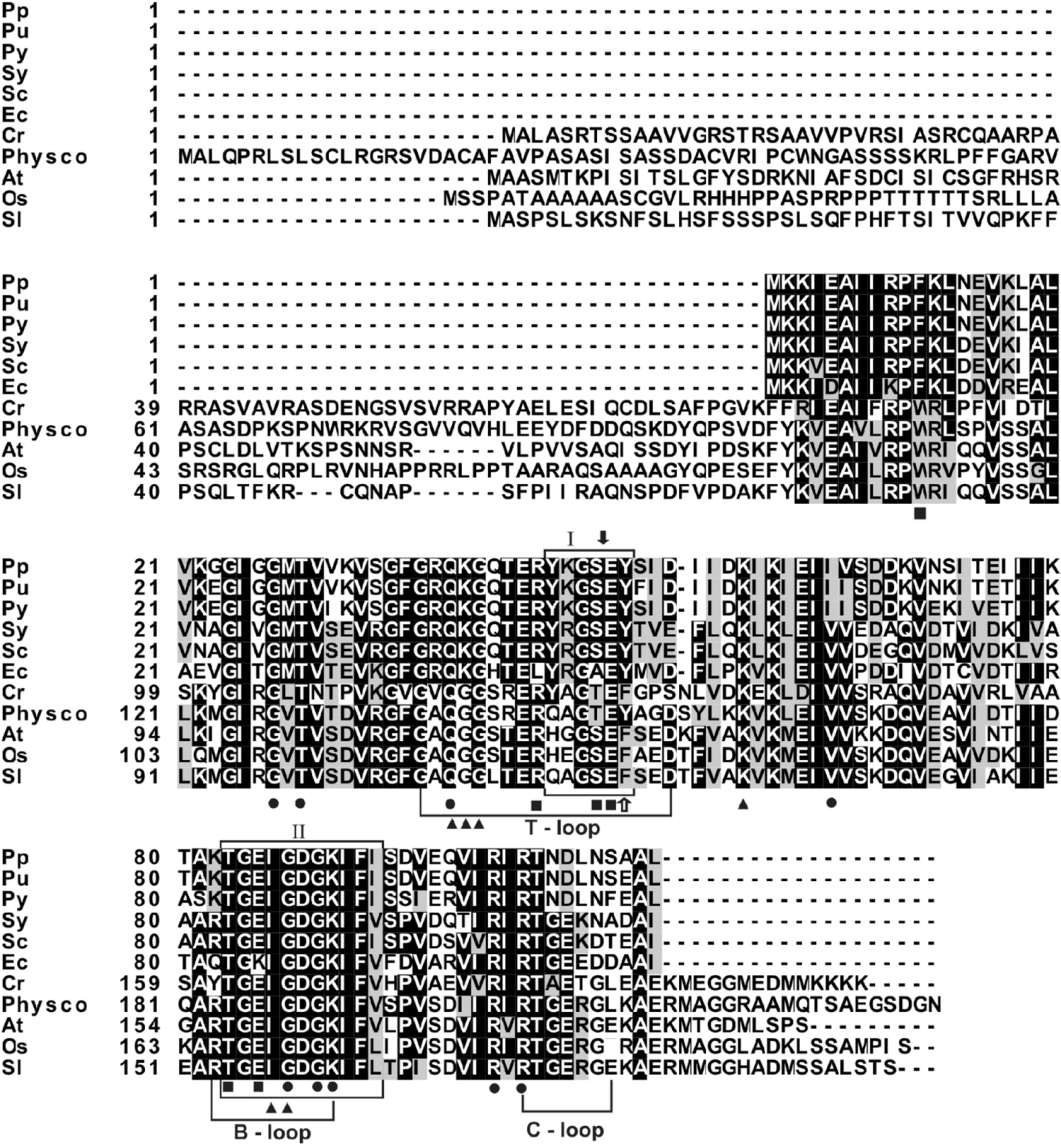
Comparison of the deduced amino acid sequences of PII polypeptides from red algae, bacteria, and Chloroplastida (green algae and higher plants). Aligned are deduced PII protein sequences from red algae: *Porphyra purpurea* (Pp; NP_053864.1), *Porphyra umbilicalis* (Pu; AFC39923.1), and *Pyropia yezoensis* (Py; AGH27579.1), bacteria: *Synechococcus elongatus* PCC 7942 (Sy; P0A3F4.1), *Synechocystis* sp. PCC 6803 (Sc; CAA66127.1), and *E. coli* (Ec; CAQ32926.1.), green algae: *Chlamydomonas reinhardtii* (Cr; A8JI83), and higher plants: *Physcomitrella patens* (Physco; BAF36548.1)*, Arabidopsis thaliana* (At; NP_192099.1), *Oryza sativa Japonica* (Os, NP_001054562.1), and *Solanum lycopersicum* (Sl, AAR14689.1). Residues highlighted in black are identical or conserved in at least 55% of all aligned PII proteins. Amino acids in a gray background represent similar residues. Box I and box II refer to PII signature patterns I and II, respectively. The positions of the Tyr residue that is uridylylated in *E. coli* PII and Ser residue that is phosphorylated in *Synechoccocus* PCC 7942 are indicated by white and solid black arrows, respectively. ATP-(●), NAGK- (■) and 2-OG-binding residues (▲) are highlighted. Alignments were made with the ClustalW program and refined manually.

The predicted full-length PpPII polypeptide (P51254) encoded by the *P. purpurea GlnB* gene has the typical length bacterial PII proteins (112 amino acids), corresponding to a molecular weight of 12.320 kDa. A sequence alignment with PII proteins from other red algae, bacteria and Chloroplastida is shown in Fig. 1. The highest degree of identity occurs with the cyanobacterial homologues *S. elongatus* PCC 7942 (63.96%) and *Synechocystis* sp. PCC 6803 (62.26%). The PpPII identity with plant PII proteins was lower and demonstrated 45.37% (*C. reinhardtii*), 52.29% (*Arabidopsis thaliana* and *Oryza sativa Japonica*) and 51.38% (*P. patens* and *Solanum lycopersicum*). Similar to PII homologs of bacteria, PpPII does not contain the N-terminal transit signal peptides and the unique C-terminal segment that are present in proteins of Chloroplastida^24^ (Fig. 1).

Regions of extremely high local identities are known as PII signature patterns that have been defined at PROSITE PS00496 and PS00638 (Fig. 1). The signature pattern I (Y-[KR]-G-[AS]-[AE]-Y) contains the residues that are involved in the interaction with NAGK. The second pattern which was found in all prokaryotic PII-proteins comprises the B-loop region [ST]-x(3)-G-[DY]-G-[KR]-[IV]-[FW]-[LIVM], and is also conserved in PpPII. This motif is involved in the binding of the phosphates of adenyl nucleotides in the effector molecule binding cleft of the PII trimers. Examination of the aligned sequences shows that residues necessary for effector molecule binding are conserved. However, since the detailed binding characteristics cannot be predicted from the mere presence of conserved residues, the effector-binding properties should be analysed.

### Binding of effectors molecular to PpPII protein

To gain additional insights into the ligand binding properties of PpPII in comparison to cyanobacterial and chloroplastid PII proteins, isothermal titration calorimetry (ITC) experiments were performed using highly purified Strep-tagged or His-tagged recombinant PpPII, SyPII, CrPII, and PhyscoPII proteins (Supplementary Fig. S1) with different protein/ligand/ concentrations to determine the best fitting conditions of the binding model. The titration experiments were repeated with different protein preparations to confirm the reproducibility of the results. The raw isothermal data were fitted into one-binding site as well as three-sequential binding sites to define the best fitting model. Fitting using a one binding site model states an average *K*_*d*_ value for all available binding sites and the mean of the stoichiometry of bound ligands. However, it was reported before that the optimal fitting for different members of PII superfamily was obtained only when using a sequential binding sites model with defining three consecutives binding sites^9,29^.

**Figure 2:**
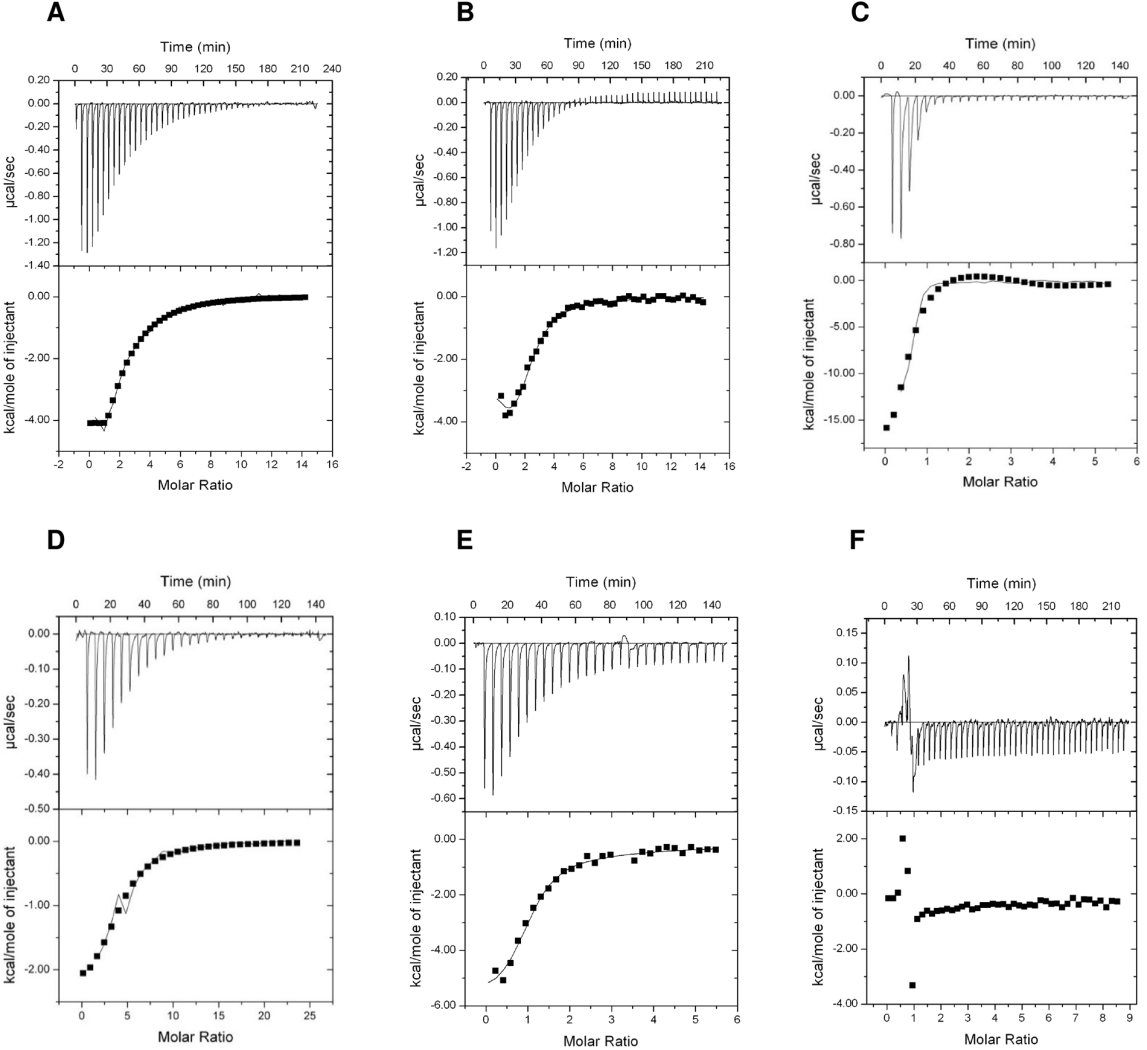
ITC analysis of binding small effector molecules to red algal PpPII protein. The upper panels show the raw data in the form of the heat effect during the titration of PpPII solution (trimer concentration) with ligands. The lower panels show the binding isotherm and the best-fit curve according to the three sequential binding sites model. Titration of PpPII protein (**A**) In 28.3 µM with 2 mM ATP. (**B**) In 28.3 µM with 2 mM ADP. (**C**) In 20 µM with 0.5 mM ATP in presence of 1 mM 2-OG. (**D**) In 9 µM with 1 mM ADP in presence of 1 mM 2-OG. (**E**) In 23.3 µM with 1 mM 2-OG in the presence of 1 mM ATP. (**F**) In 23.3 µM with 1 mM 2-OG in the presence of 1 mM ADP.

Table 1 and Fig. 2 show the dissociation constants and the binding isotherms for binding of the small effector molecules to red algal PpPII protein. Under optimal binding conditions, robust binding of ATP and ADP was detectable. As shown in Fig. 2A and B, PpPII protein exhibited high affinity toward ATP and ADP in µM range. When the data are fitted according to a model assuming independent binding sites, the average *K*_*d*_ value for all binding sites of PpPII protein bound to ATP or ADP were about 22.0 and 7.2 µM, respectively, and with a respective stoichiometry of 2.5 and 2.7 per PII trimer, respectively. Data fitting using three sequential binding sites model revealed two high-affinity sites (sites one and two) and a third low-affinity site. This analysis resolved a low *K*_*d1*_ value for the first binding site of 0.5 µM for ATP, and 3.4 µM for ADP. For binding site 2, the *K*_*d2*_ values were 18.7 µM for ATP and 15.2 µM for ADP, and for binding sites 3, *K*_*d3*_ of 141.6 µM for ATP and 131.8 µM for ADP (Table 1). The binding affinities towards ATP and ADP are very similar, reflecting that the ADP binds to the algal PpPII protein almost as efficient as ATP.

**Table 1:** Dissociation constants (*K*_*d*_) for ATP, ADP and 2-OG as indicated binding to recombinant red algal PpPII and cyanobacterial SyPII proteins, respectively.

Titrant/Protein	One-site binding model		Three-sites binding model		
	Average *K*_*d*_ (µM)	N^*^	*K*_*d1*_ (µM)	*K*_*d2*_ (µM)	*K*_*d3*_ (µM)
**Red algal PpPII**					
ATP	22.0 ± 10.8	2.5	0.5 ± 0.2	18.7 ± 9.2	141.6 ± 80.8
ADP	7.2 ± 2.5	2.7	3.4 ± 0.5	15.2 ± 0.7	131.8 ± 2.7
ATP (in presence 2-OG)	0.2 ± 0.3	1.0	1.4 ± 1.8	1.0 ± 0.9	83.9 ± 62.4
ADP (in presence 2-OG)	10.8 ± 2.8	2.7	10.3 ± 10.0	43.6 ± 4.7	125.9 ± 0.9
2-OG (in presence ATP)	4.0 ± 2.4	1.0	2.7 ± 1.4	50.7 ± 16.1	80.0 ± 22.0
2-OG (in presence ADP)	No binding				
**Cyanobacterial SyPII**					
ATP	43.3 ± 37.7	3.2	7.5 ± 7.3	15.9 ± 12.2	85.4 ± 74.6
ADP^9^	NF	NF	(10.6 ± 3.2)	(19.3 ± 2.3)	(133.4 ± 5.2)
ATP (in presence 2-OG)	2.7 ± 0.1	1.8	0.5 ± 0.5	3.0 ± 1.7	325.5 ± 171.0
ADP (in presence 2-OG and 150 µM ADP)	45.8 ± 44.0	4.0	10.7 ± 1.9	26.4 ± 4.2	100.9 ± 102.5
2-OG (in presence ATP)	21.1 ± 6.8	1.4	2.9 ± 4.0	6.4 ± 2.7	83.0 ± 43.7
2-OG (in presence ADP)^49^	(No binding)				

The raw data were fitted using one-site and three sequential binding sites models for PII trimer. The *K*_*d*_ values correspond to the mean of the independent experiments ± SD. For comparison, data for ADP and 2-OG in presence of ADP binding to SyPII are given in parentheses. *N, number of calculated binding sites; NF, not fitted.

Both, binding of ATP and ADP, display a strong anticooperativity among the three consecutive sites. Compared to the adenyl nucleotide binding properties of the previously analysed cyanobacterial SyPII protein^9^, the overall characteristics are similar. However, in the PpPII protein, the extent of anticooperativity is even higher, spanning a range of more than two orders of magnitude, whereas in SyPII, the range is only one order of magnitude (from 4 µM to 47 µM for ATP and from 10 to 133 µM for ADP)^9^. For binding site one, there is a clear preference for ATP, whereas in the other two sites, competition between ATP and ADP will occur.

For 2-OG binding to PpPII protein, strong binding isotherm was noticeable in the presence of ATP (Fig. 2E). Optimal fitting was obtained assuming three sequential binding sites model, again revealing strong anti-cooperativity. The first site was occupied at low 2-OG concentrations of *K*_*d1*_ 2.7 µM, the second site requires approximately 19-fold higher concentrations of 2-OG, corresponding to a *K*_*d2*_ of 50.7 μM. Occupation of the third binding site took place with a *K*_*d3*_ of 80.0 μM. These values are quite similar to the properties of cyanobacterial SyPII^9^. No binding of 2-OG could be detected in presence of ADP, in contrast to what was reported for plant PII protein from *A. thaliana*^30^.

Next, we investigated the influence of 2-OG on the binding of ATP or ADP to PpPII. As shown for several bacterial PII proteins, the presence of 2-OG highly increased the affinity towards ATP binding (Table 1 and Fig. 2C). By contrast, in the presence of 2-OG the binding enthalpy for ADP was strongly decreased when compared with the titration in the absence of 2-OG (Fig. 2, compare D with B). Therefore, the presence of 2-OG increased the *K*_*d*_ values of the first and the second ADP binding sites (Table 1).

**Figure 3:**
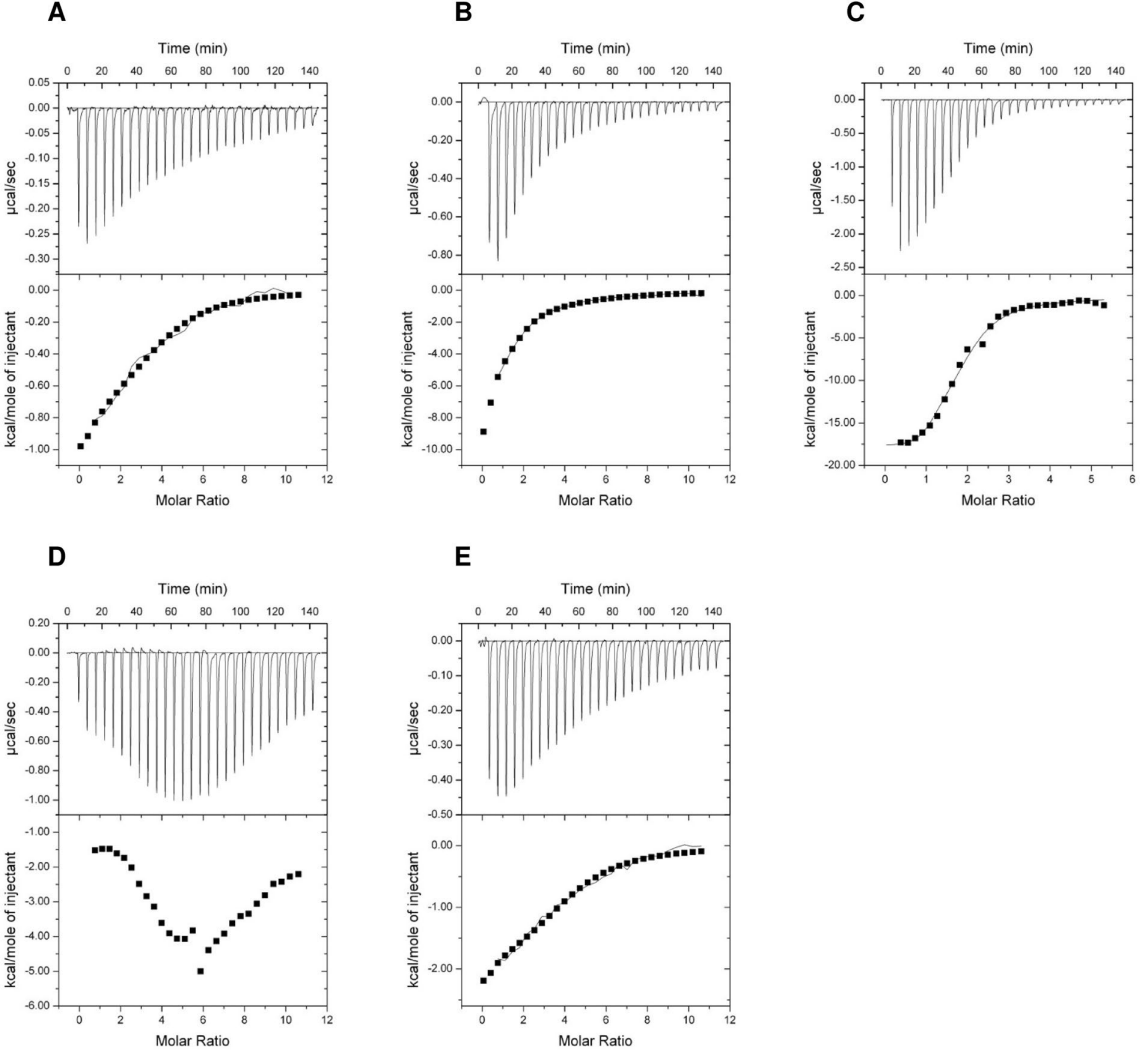
ITC analysis of binding small effector molecules to cyanobacterial SyPII protein. The upper panels show the raw data in the form of the heat effect during the titration of SyPII solution (trimer concentration) with ligands. The lower panels show the binding isotherm and the best-fit curve according to the three sequential binding sites model. Titration of SyPII protein (**A**) In 20 µM with 1 mM ATP. (**B**) In 20 µM with 1 mM 2-OG in the presence of 2 mM ATP. (**C**) In 20 µM with 0.5 mM ATP in presence of 1 mM 2-OG. (**D**) In 20 µM with 1 mM ADP in presence of 1 mM 2-OG. (**E**) In 20 µM with 1 mM ADP in the presence of 1 mM 2-OG and 150 µM ADP.

To study comparatively the sensing properties of PII proteins in the course of the endosymbiont (chloroplast) evolution, we choose the PII protein from the cyanobacterium *S. elongatus* (SyPII) (Table 1), from the green alga *C. reinhardtii* (CrPII) and from the higher eukaryotic moss-plant *P. patens* (PhyscoPII) (Table 2). As reported previously, SyPII protein was able to bind ATP, ADP and 2-OG, with 2-OG being bound exclusively in the presence of ATP with negative cooperativity for the three-available binding sites^9,31^. As a control, we obtained comparable results with the previously reported *K*_*d*_ values for ATP and 2-OG binding in presence of ATP (Table 1, Fig. 3A and B)^9^. Moreover, in the presence of 2-OG the binding enthalpy for ATP was strongly increased as compared to binding of ATP alone (Fig. 3, compare C with A). Thus, like in other PII proteins, the presence of 2-OG enhances the affinity for ATP (Table 1). Furthermore, the effect of 2-OG against ADP on SyPII binding was investigated. First, ADP was titrated to SyPII protein in the presence of 1 mM 2-OG. The titration curves showed a complex biphasic curve; in the first injections, the enthalpy increased, until a maximum was reached at ADP concentration of 80.0 ± 7.1 µM (Fig. 3D). Subsequently, the heat change signals decreased again, indicating gradual saturation of PII by ADP (Fig. 3D). Therefore, it seems that elevated 2-OG levels antagonistically prevent formation of the PII:ADP complex. To test whether a certain threshold level of ADP can relieve the inhibitory effect of 2-OG on ADP binding to SyPII (suggested by the biphasic curve), ADP was titrated again against SyPII protein in presence of 1 mM 2-OG and 150 µM ADP (Fig. 3E). A typical curve was obtained again which could be fitted with sequential binding (Table 1).

**Table 2:** Dissociation constants (*K*_*d*_) for ATP, ADP and 2-OG as indicated binding to recombinant green algal CrPII and plant-moss PhyscoPII proteins, respectively.

Titrant/Protein	One-site binding model		Three-sites binding model		
	Average *K*_*d*_ (µM)	N^*^	*K*_*d1*_ (µM)	*K*_*d2*_ (µM)	*K*_*d3*_ (µM)
**Green algal CrPII**					
ATP	No binding				
ADP	No binding				
ATP (in presence 2-OG)	35.8 ± 1.6	2.6	5.8 ± 5.8	53.1 ± 58.3	61.6 ± 9.7
ADP (in presence 2-OG)	No binding				
2-OG (in presence ATP)	15.0 ± 5.4	2.6	4.2 ± 0.7	21.3 ± 11.0	45.5 ± 19.8
2-OG (in presence ADP)	No binding				
**Plant-moss PhyscoPII**					
ATP	26.8 ± 10.5	2.9	5.7 ± 1.4	13.6 ± 7.2	48.2 ± 29.3
ADP	No binding				
ATP (in presence 2-OG)	17.6 ± 7.5	5.3	8.0 ± 6.0	16.3 ± 11.4	128.8 ± 128.1
ADP (in presence 2-OG)	No binding				
2-OG (in presence ATP)	No binding				

The raw data were fitted using one-site and three sequential binding sites models for PII trimer. The *K*_*d*_ values correspond to the mean of the independent experiments ± SD. *N, number of calculated binding sites.

**Figure 4:**
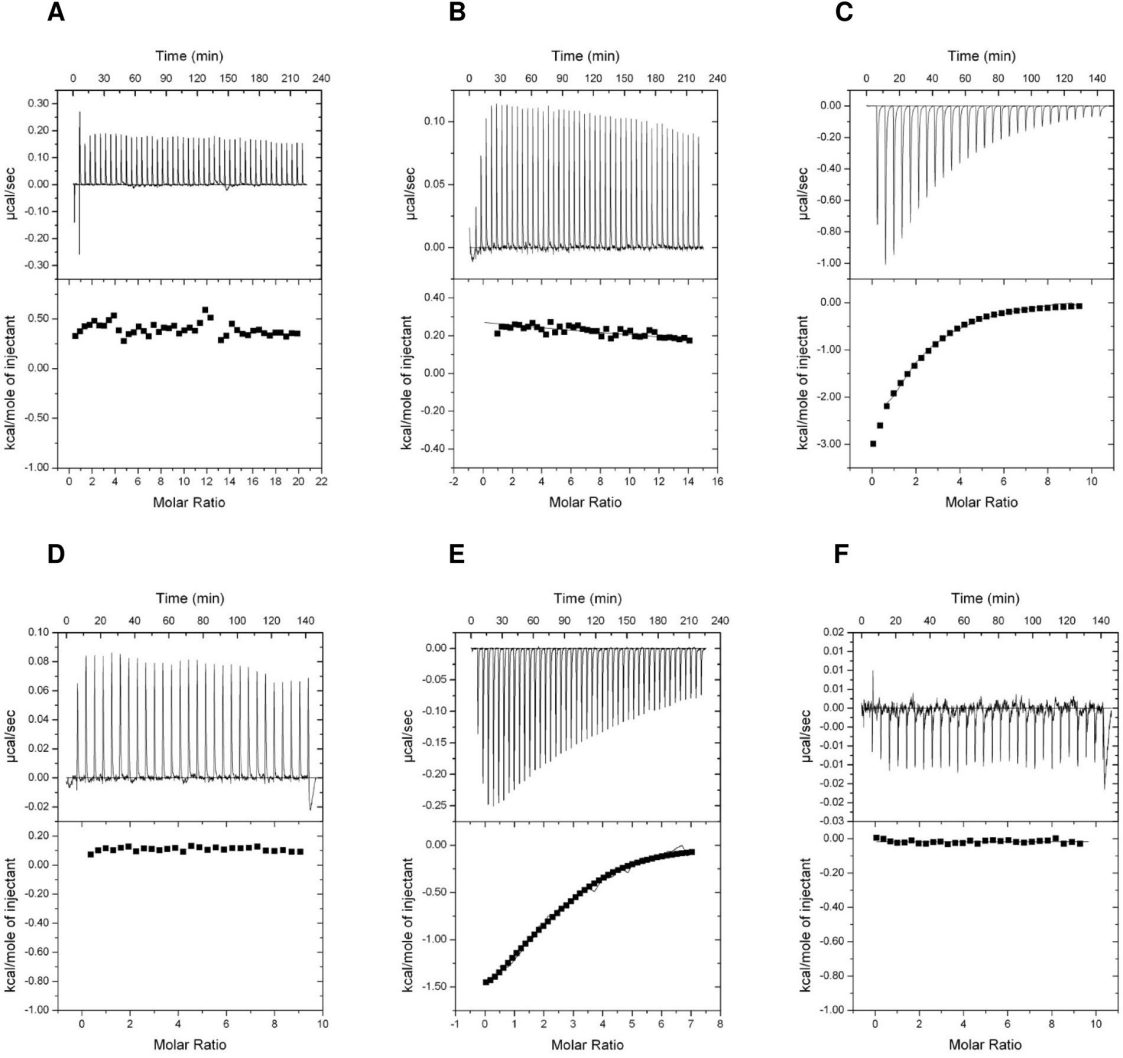
ITC analysis of binding small effector molecules to green algal CrPII protein. The upper panels show the raw data in the form of the heat effect during the titration of CrPII solution (trimer concentration) with ligands. The lower panels show the binding isotherm and the best-fit curve according to the three sequential binding sites model. Titration of CrPII protein (**A**) In 20 µM with 2 mM ATP. (**B**) In 28.3 µM with 2 mM ADP. (**C**) In 45 µM with 2 mM ATP in presence of 2 mM 2-OG. (**D**) In 45 µM with 2 mM ADP in presence of 2 mM 2-OG. (**E**) In 28.3 µM with 1 mM 2-OG in the presence of 2 mM ATP. (**F**) In 44 µM with 1 mM 2-OG in the presence of 2 mM ADP.

Table 2 and Fig. 4 show the dissociation constants and the binding isotherms for the binding of the small effector molecules to green algal CrPII protein. Interestingly, we were unable to notice any binding towards ATP and ADP (Fig. 4A and B). However, the crystal structure of CrPII in complex with ATP and 2-OG was solved previously (PDB: 4USI)^24^. Therefore, we tested ATP binding in the presence of 2-OG, which revealed a strong ATP binding with high affinity in µM range (Fig. 4C). This suggests that the binding affinities toward ATP under the tested conditions are very low and the ATP affinities increase dramatically in presence of 2-OG. As expected, no ADP binding to CrPII protein was detected in presence of 2-OG (Fig. 4D). Conversely, we observe binding of 2-OG in presence of ATP but never observed binding of 2-OG in presence of ADP (Fig. 4E and F).

**Figure 5:**
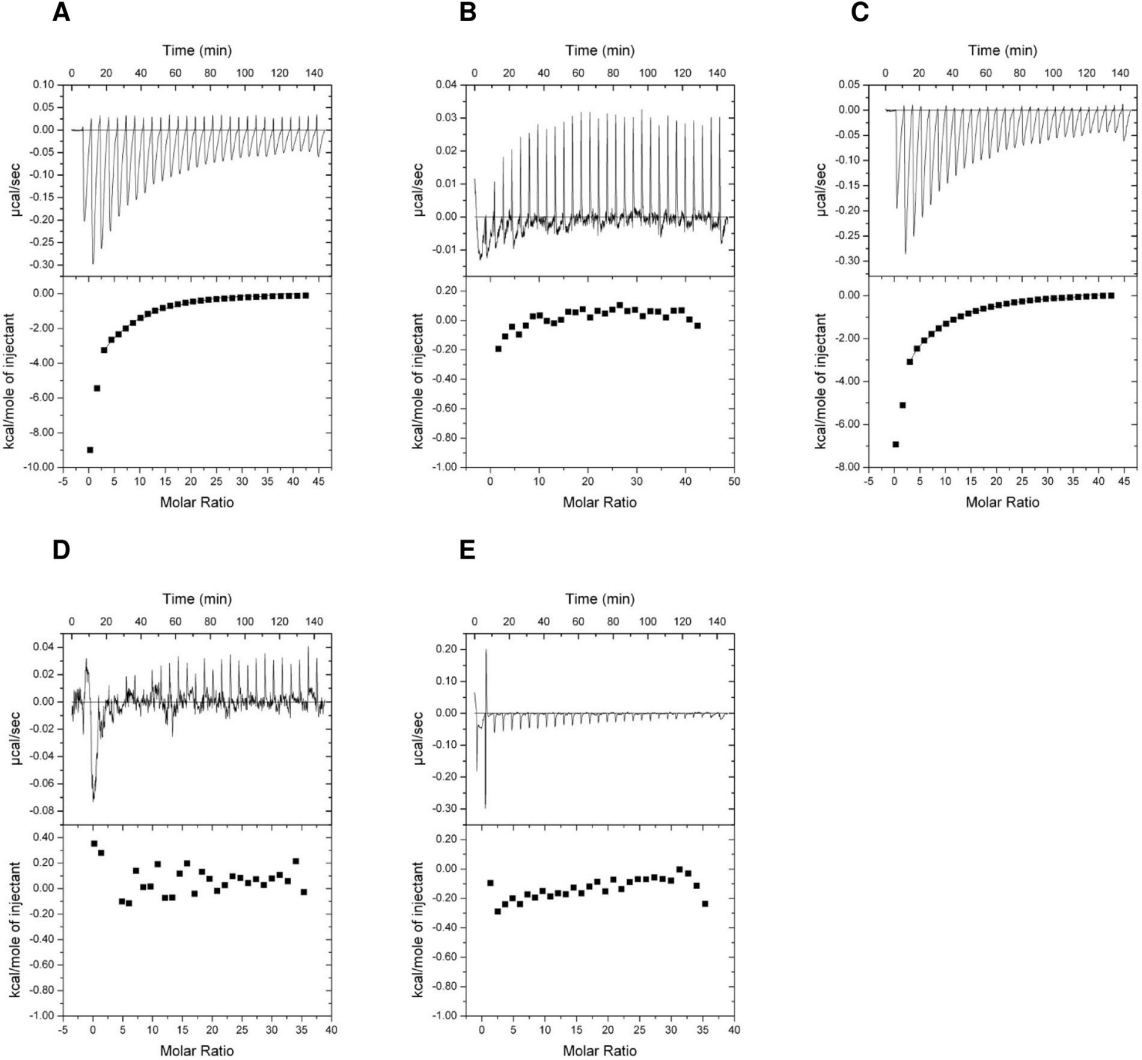
ITC analysis of binding small effector molecules to plant-moss PhyscoPII protein. The upper panels show the raw data in the form of the heat effect during the titration of PhyscoPII solution (trimer concentration) with ligands. The lower panels show the binding isotherm and the best-fit curve according to the three sequential binding sites model. Titration of PhyscoPII (**A**) In 5 µM with 1 mM ATP. (**B**) In 5 µM with 1 mM ADP. (**C**) In 5 µM with 1 mM ATP in presence of 2 mM 2-OG. (**D**) In 6 µM with 1 mM ADP in presence of 1 mM 2-OG. (**E**) In 6 µM with 1 mM 2-OG in the presence of 1 mM ATP.

Finally, we assayed the plant PII protein from moss *P. patens* (PhyscoPII) for binding of ATP, ADP and 2-OG. We were able to detect ATP binding (Table 2, Fig. 5A). Data fitting using three sequential binding sites model revealed high-affinity for the first binding site (*K*_*d*_ of 5.7 µM) and anticooperativeity on the second and third sites with *K*_*d*_ values of 13.6 and 48.2 µM, respectively. Surprisingly, we were unable to detect any binding for ADP, nor for 2-OG in the presence of ATP (Table 2, Fig. 5B,D and E). Consistent with this, the presence of 2-OG did not change the affinity for ATP (Table 2, and Fig. 5C). Thus, unlike the other PII proteins, 2-OG seems to have no effect on the PhyscoPII protein.

### Enzymatic properties of the NAGK-PpPII complex

**Figure 6:**
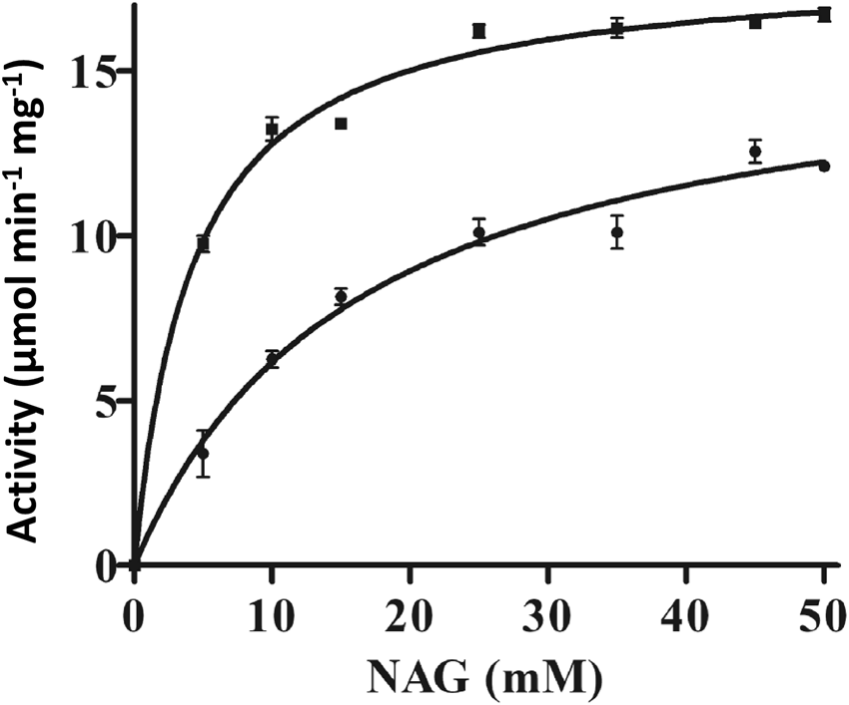
The catalytic activity of ScNAGK with or without PpPII protein. NAG was used as a variable substrate at fixed 10 mM ATP. The curves were fitted with GraphPad prism software. Standard deviations from triplicate experiments are indicated by error bars. (●), free ScNAGK; (■), ScNAGK with PpPII.

PpPII shared the signature residues involved in NAGK-PII interaction (Fig. 1). The experiments shown above (Table 1, Fig. 2) indicated that the PpPII protein responded to the effector molecules ATP, ADP and 2-OG quite similarly to cyanobacterial PII. Since, the NAGK is highly conserved enzyme across different domains of life. The amino acid sequences of NAGK enzymes are sharing high identity across different phyla (Supplementary Fig. S2). Earlier, we showed that the signal transduction PII proteins across different domains of life able to regulate NAGK activity of different phyla^32^. Therefore, we wanted subsequently to investigate the putative interaction between the red algal PpPII and cyanobacterial NAGK, and the ability of PpPII protein to activate NAGK. A recombinant NAGK from *Synechocystis* sp. PCC 6803 (ScNAGK) was expressed with its N-terminus fused to a His_6_ tag. The kinetic constants of the purified recombinant ScNAGK enzyme showed a *K*_*m*_ value for N-acetyl glutamate (NAG) of 16.23 ± 2.14 mM and a *K*_*cat*_ of 8.93 ± 1.40 s^−1^. In the presence of PpPII (Fig. 6), the apparent *K*_*m*_ for NAG dropped to 4.25 ± 0.36, whereas the *K*_*cat*_ remained almost unchanged with a value to 10.02 ± 1.11 s^−1^. This corresponds to a catalytic efficiency (*K*_*cat*_/*K*_*m*_) of 0.55 × 10^3^ and 2.36 × 10^3^ s^−1^M^−1^ for free and PpPII-complexed ScNAGK, respectively. The data showed that complex formation with PpPII caused an about 4-fold increase of the overall NAGK catalytic efficiency.

### Biochemical characterization of interaction between PpPII and ScNAGK

**Figure 7:**
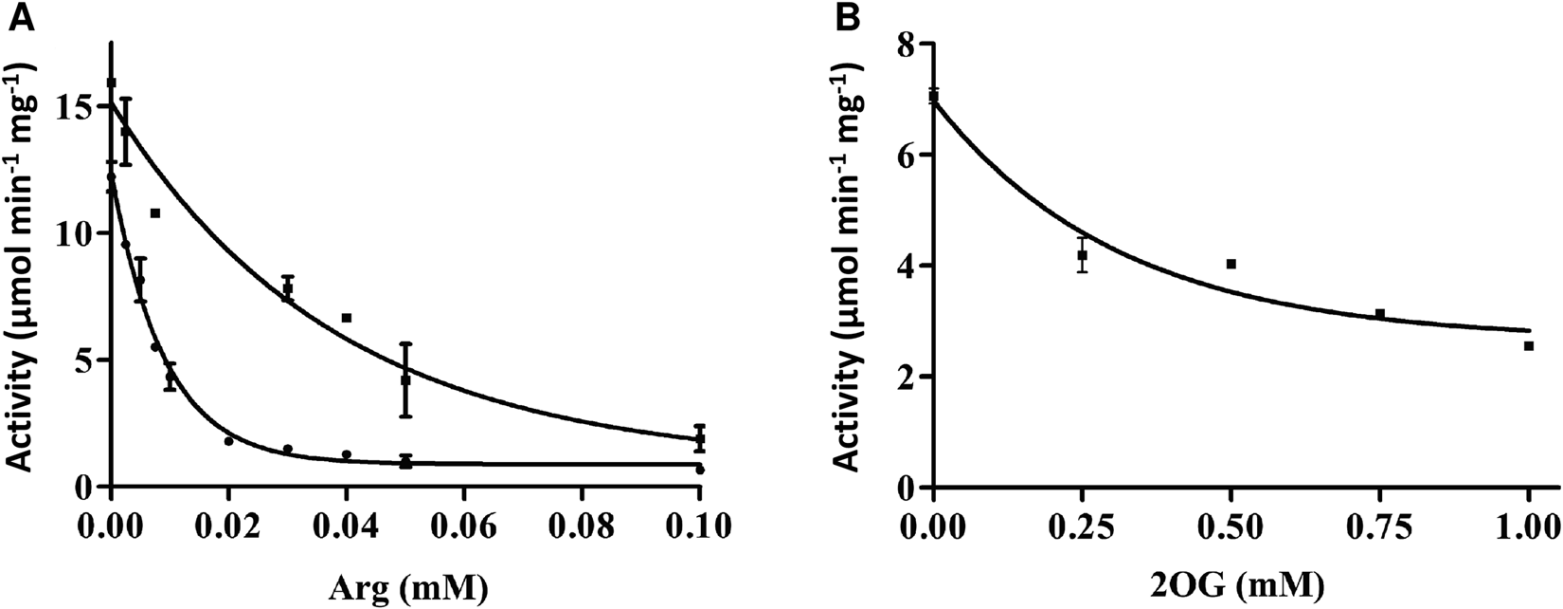
Arginine inhibition and antagonistic effect of 2-OG on NAGK activation by PpPII in the presence of arginine. (**A**) Inhibition of ScNAGK activity by arginine inhibition of ScNAGK in the absence (●) or presence of PpPII (■). Coupled NAGK assays were performed in the presence of 50 mM NAG and 10 mM ATP, together with increasing concentrations of arginine, as indicated. (**B**) Effect of 2-OG on PpPII activation of ScNAGK. Assays contained 50 mM NAG, 10 mM ATP, and 0.01 mM arginine, together with increasing concentrations of 2-OG, as indicated. Standard deviations from triplicate experiments are indicated by error bars.

The relief from arginine inhibition by PII-NAGK complex formation is the major checkpoint for metabolic control of the arginine biosynthesis pathway in cyanobacteria and Chloroplastida^15,24^. To determine whether PpPII affected the catalytic activity of ScNAGK, we assayed NAGK by a coupled assay^32^. Feedback inhibition by arginine occurred with a half maximal inhibitory concentration (IC_50_) of 6.2 µM (Fig. 7A). Addition of PpPII protein to ScNAGK raised the IC_50_ for arginine by approximately 4-fold. As expected, the presence of PpPII changed arginine inhibition of ScNAGK without the presence of glutamine.

Like other canonical PII proteins, PpPII sensed 2-OG (Fig. 2). In cyanobacteria and plants, binding of 2-OG usually antagonizes the interaction of PII with NAGK. To determine next the response of PpPII-ScNAGK interaction towards 2-OG, an assay was set up, where PpPII and ScNAGK (at a ratio of 5 PII trimers to one NAGK hexamer) were incubated together with arginine and titrated with increasing concentrations of 2-OG. In these assays, the mixture contained 0.01 mM arginine that shows relatively low inhibition for the PpPII-ScNAGK complex but already processes highly inhibitory for free ScNAGK (Fig. 7A). As shown in Fig. 7B, addition of 2-OG to the PpPII–ScNAGK–arginine mixture indeed inhibited ScNAGK activity in a concentration-dependent manner. Half-maximal inhibition (IC_50_) of NAGK activity was attained at 0.22 ± 0.01 mM 2-OG. This concentration additionally reflected the affinity of PpPII towards the effector molecule 2-OG and indicates that all three binding sites have to be occupied by 2-OG in order to abrogate the productive interaction with NAGK.

### Competition between ATP and ADP at different ratios affects PpPII-mediated activation of NAGK

**Figure 8:**
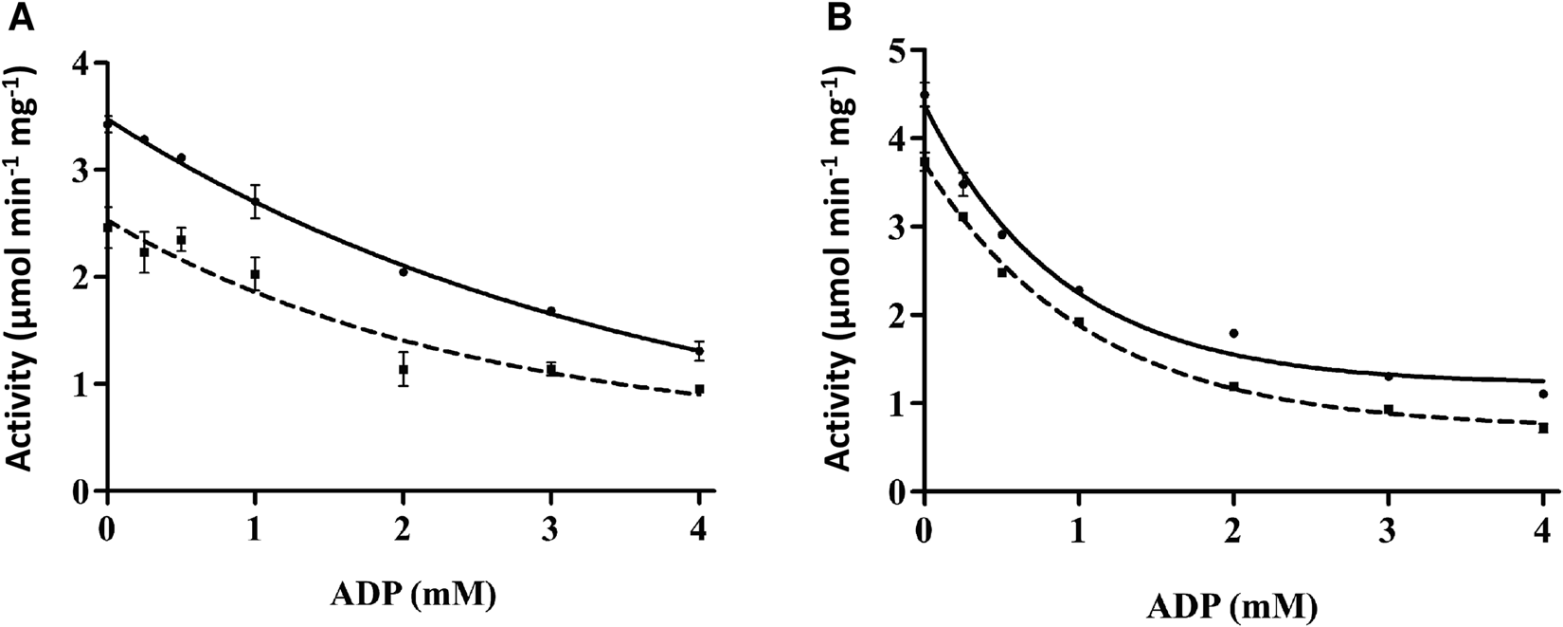
Effect of ADP on PpPII-mediated NAGK activity in the AGPR-coupled assay. Assays were performed in the presence of ATP at a concentration of 1 mM (dashed line) or 2 mM (continuous line) as indicated. Enzyme activity without (**A**) or with (**B**) PpPII protein. The curves were fitted with GraphPad prism software. Standard deviations from triplicate experiments are indicated by error bars.

All available structural information of PII proteins revelas that ATP and ADP bind to the same binding sites^2,7^. When ATP and ADP are simultaneously present, they will compete for binding to the PII trimer^31^. Since, the ATP/ADP binding site of the red algal PpPII protein is highly conserved (Fig. 1), competition between the nucleotides will occur. Since the activation of NAGK by PII is dependent on the ATP-ligated state of PII^9^ and ADP inhibits PII-NAGK complex formation^9,31^, competition between ATP/ADP for PII binding can be monitored by measuring NAGK activity^31^ under different ATP/ADP ratios. For this assay, we used an AGPR-coupled activity assay for NAGK^33^, which allows measurement of NAGK activity in presence of ADP. Different concentrations of ADP were added to reaction mixtures containing either 1 mM or 2 mM ATP, in the absence or presence of PpPII (Fig. 8). Addition of ADP led to a monotonical and shallow decrease in the activity of ScNAGK at both fixed ATP concentration. In the presence of PpPII, the effect by the addition of ADP was more pronounced (Fig. 8B). A steep decrease was observed up to 2 mM ADP, indicating that complex formation with NAGK is sensitive towards elevated ADP levels.

At the highest ADP/ATP ratio (4 mM ADP with 1 mM ATP), ScNAGK activity in the presence of PpPII was as low as ScNAGK activity in the absence of PpPII (Fig. 8). These responses were very similar to those of *S. elongatus* NAGK-PII complex to variable ATP/ADP levels^31^.

### Investigation of the PipX-PpPII interaction

In cyanobacteria, PipX is the second known receptor of PII signaling^18^. We questioned whether red algal PII proteins have lost or retained the ability to bind to PipX. To test this possibility, we first analyzed complex formation using pull-down experiments (Supplementary Fig. S3A and B). The PII protein from *Synechocystis* sp. PCC 6803 (ScPII) was mixed with PipX in the presence of ADP and then the Strep-tagged PII was immobilized on the Strep-Tactin II column (Supplementary Fig. S3A). After extensive washes, the proteins were eluted with desthiobiotin and analyzed by Tricine-SDS-PAGE. Both proteins were detected in the elution. Thus, confirming previous studies^16–18^, cyanobacterial PII was bound to PipX. When PpPII in the presence of PipX was immobilized on the column, no PipX signal was detected in the elution (Supplementary Fig. S3B). This experiment showed that PpPII did not interact with PipX.

Additionally, we assessed whether PpPII could bind to PipX by surface plasmon resonance (SPR) spectroscopy (Supplementary Fig. S3C). The N-terminally His-tagged ScPipX was immobilized on a Ni-HTG sensor chip and probed with ScPII together with effector molecule ADP. Interaction between PpPII and PipX was not observed. These results supported the view that cyanobacterial PipX was not the target of PpPII.

## Discussion

During the evolution of Rhodophyta, the PII proteins that are ubiquitously present in Bacteria, Archaea and in the chloroplasts of green algae and land plants^1,5,23^ have been lost in most red algae. The Rhodophyta are considered to be a member of the founding lineage of photosynthetic eukaryotes (also known as Archaeplastida), whose progenitors captured the ancestral cyanobacterium-derived plastid^34,35^. Among the red algae, genes encoding putative PII proteins have only been identified until now in *P. purpurea, P. umbilicalis* and *P. yezoensis*^20,21^. In this work, PII from *P. purpurea* is characterized with respect to its functional similarities and differences compared with PII proteins from cyanobacteria and chloroplastida.

Like their bacterial PII homologs, the red algal PII proteins do not have the transit signal N- and the unique C-terminal sequences present in the green algal and land plant proteins of this family (Fig. 1). In addition, the sequences of PII from the cyanobacteria and from *P. purpurea, P. umbilicalis* and *P. yezoensis* are highly similar. All known PII proteins are able to sense and integrate signals from central metabolism: competitive binding of ADP and ATP reflects the energy state and binding of 2-OG indicates the cellular C-N balance^36–38^. The PII trimers contain three effector nucleotide-binding sites, one in each intersubunit cleft^9^. The PpPII protein of *P. purpurea* also binds three molecules of ATP and ADP, both with negative cooperativity, and with ATP having slightly higher affinity than ADP (Fig. 2A and B and Table 1), similar to the PII protein of *S. elongatus*^9^. In the presence of Mg^2+^-ATP, the three sites can also bind 2-OG (Fig. 2E). The three 2-OG binding sites exhibit negative cooperativity to each other (Table 1) but the binding of ATP and 2-OG is synergistic to each other. This synergy of ATP and 2-OG binding is typical of bacterial PII proteins^30,36,39–41^. Conversely, 2-OG quenches binding of ADP to PpPII (Fig. 2D).

For green algal CrPII protein, we were unable to detect any binding for ATP alone, while in presence of 2-OG a strong binding isotherm for ATP was restored. This indicates that the affinity of CrPII protein towards ATP is below detection limit and 2-OG is required to constitute an efficient binding site for ATP. In the case of PhyscoPII, ATP binding follows the ancestral (cyanobacterial) mode. However, we were unable to detect ADP binding for CrPII as well as for PhyscoPII protein. The loss of ADP binding suggests a differentiation in the energy sensing role of PII proteins in chloroplast evolution. Whereas in cyanobacteria and red algae, binding of ATP and ADP is competitive, the Chloroplastida seem to have specialized the PII function towards the ATP-ligated state. Furthermore, the lack of 2-OG response in PhyscoPII protein was striking. It suggests the regulatory role of 2-OG could have become obsolete in some lineages, and may be completely replaced by the later acquired glutamine sensing properties of PII proteins: green plant PII proteins are able to sense glutamine through a small C-terminal extension (termed Q-Loop) which represents a low-affinity glutamine binding site^24^. A previous publication reported that *Arabidopsis* PII protein binds 2-OG in presence on ADP^30^, which contrasts the results reported here. Since *Arabidopsis* is an exception in the plant kingdom due to its secondary loss of the glutamine binding site^24^, it may have compensated this loss by an enhanced affinity towards 2-OG. In some prokaryotic lineages, PII proteins were reported that lack 2-OG responses such as the protein form archaeon *Archaeoglobus fulgidus*^42^.

Interestingly for ADP-binding PII proteins (red algal PpPII and cyanobacterial SyPII), the pre-equilibration of the proteins with 2-OG lowers the ADP affinities in ITC measurements, revealing strong antagonistic effect between 2-OG and ADP binding, especially in case of cyanobacterial SyPII protein, which shows complex ITC curve (Fig. 3D). The antagonistic effect of 2-OG on ADP binding affinities was mentioned previously for *A. thaliana* PII^30^ and explained mechanistically for *E. coli* PII protein^37^. Modeling the complex binding possibilities of PII proteins provided a mathematical interpretation of the synergetic effect between 2-OG/ATP and antagonistic effect between 2-OG/ADP. Under high physiological concentrations of 2-OG, 2-OG antagonizes ADP binding to PII by favoring the binding of the competing nucleotide ATP, since the presence of 2-OG increases the number of PII:ATP complex states^43^. Together, this demonstrates that 2-OG shifts the competition of ATP and ADP for PII binding in favor of ATP to ensure formation of the final PII:ATP:2-OG complex under high 2-OG conditions.

Altogether, the comparative analysis of PII binding properties points out that the red algal PpPII protein is quite distinct from plant PII proteins, and clearly closer to cyanobacterial PII proteins. In the blue and red lineages, 2-OG is strongly synergistic with ATP binding to PII, while antagonistic for ADP binding. In later stages of evolution of Viridiplantae, the PII proteins diverged in their properties, becoming very heterogeneous with respect to 2-OG and to ADP binding, while only the binding of ATP in presence of 2-OG has been conserved.

The interaction between PII proteins and NAGK was shown to be highly conserved from cyanobacteria to higher plants^24,25,32^. We showed previously that PII proteins across domains of life are able to interact with PII-controlled NAGK enzymes of different phyla^32^. In agreement, here we show that PpPII protein is able to interact and regulate *Synechocystis* sp. PCC 6803 NAGK (Fig. 6), which indicates the evolutionary conserved ability of the PII signaling protein to control NAGK activity across domains of life. When nitrogen is abundant, oxygenic phototrophic organisms are suggested to store nitrogen as arginine, by relieving feedback inhibition of the arginine biosynthesis controlling enzyme, NAGK^44,45^. The C-terminus of plant PII (except *Brassicaceae*) contains an extension, which binds glutamine and mediates glutamine sensing^24^. As expected from the lack of the C-terminal extension, PpPII protein relieves arginine inhibition of ScNAGK (Fig. 7A) in a glutamine-independent manner. Moreover, PpPII-mediated relief from arginine inhibition is antagonized by 2-OG (Fig. 7B).

Apparently, NAGK from cyanobacteria highly resembles NAGK from those red algae, which possess the PII protein. Intriguingly, the residues critically involved in PII interaction, are not conserved in NAGK sequences form red algae, which have lost the *glnB* gene (encoding PII) (Supplementary Fig. S2). Of particular importance is the arginine residues, which corresponds to R233 of NAGK from *S. elongatus*. This residue forms a salt bridge with B-loop residue E85 of SyPII, which represents the first step (the encounter complex) of PII-NAGK complex formation^9,46^, Since the critical residues for compelx formation are conserved in PII and NAGK sequences of *P. purpurea* (Fig. 1 and Supplementary Fig. S2), we propose that complex formation between ScNAGK and red algal PpPII protein follows the same mechanism.

PII proteins from cyanobacteria directly sense the adenylate energy charge, resulting in target-dependent differential modification of the PII-signaling properties^8,31^. The kinetic activation of ScNAGK by PpPII was weakened by ADP (Fig. 8). Furthermore, in the absence of PpPII, ScNAGK responded only weakly to different ATP/ADP ratios, with about 1.8-fold reduction of activity comparing 0 and 4 mM ADP at any used ATP concentration. This indicates that the NAGK activity response towards different ATP/ADP ratios in presence of PpPII operates through competitive binding of the adenyle-nucleotides to the PII binding pocket. Together, the similarity in NAGK regulation between Rhodophyta and cyanobacteria suggests that nitrogen metabolism in red algal plastids has conserved main regulatory properties from the ancestral cyanobacterial endosymbiont. They use 2-OG and the energy state as the status reporters that control nitrogen storage metabolism.

The second well-studied interaction partner of PII from cyanobacteria is protein PipX^16,18^, the transcriptional co-activator of NtcA. The inability of the PpPII protein to bind ScPipX agrees with the fact that the PipX gene was not conserved in the endosymbiontic process. Due to the loss of PipX in the endosymbiont, no selective pressure forced PpPII to maintain the PipX binding properties. Consequently, we confirmed that PipX is highly specific for cyanobacterial PII protein even with conservation of the interacting residues which required for formation of PII-PipX complex among algal PII proteins. Apparently, the transcriptional machinery of the eukaryotic host took over nitrogen-responsive gene expression and, thereby, PipX became obsolete. Identification of other PII partners in red algae as well as in cyanobacteria and plants would help to further delineate details of the endosymbotic process and to better understand the driving forces in evolution that resulted in the greening of our planet.

## Materials and Methods

### Cloning, expression and purification of PII proteins

A synthetic gene Block encoding *glnB* gene of PpPII with an optimized codon usage for the *E. coli* expression was synthesized by IDT, USA. The PpPII gene was amplified using the primer pair 5′-ATGAATAGTTCGACAAAAATCTAGATAACGAGGGCAAAAAATGAAAAAGATCGAAGCTATTATTC-3′ and 5′- AAGCTTATTATTTTTCGAACTGCGGGTGGCTCCAAGCGCTAAGAGCTGCGCTGTTTAAATC-3′. The PCR products of PpPII and of *glnB* gene from *Synechocystis* sp. PCC 6803 (ScPII) were cloned into pASK-IBA3 vector (IBA, Germany) as described previously^47^. Recombinant PpPII and ScPII proteins containing a C-terminal Strep-tag II peptide were overexpressed in PII-deficient *E. coli* RB9060^48^, and purified using affinity chromatography on a Strep-Tactin Superflow column (IBA) similarly as described previously for overexpression of PII protein from *S. elongatus* PCC 7942 (SyPII)^15^. For the production of recombinant PpPII with a N-terminal His_6_-tag the synthetic gene Blocks fragment of PpPII was cloned directly into NdeI-digested pET15b vector (Novagen-Merck, Germany) by aqua cloning^49^. The recombinant N-terminal fused His_6_-tag-PpPII protein with was overexpressed in *E. coli* Rosetta (Novagen) and purified on a Ni-NTA column as in^50^. Truncated versions of recombinant PII proteins from *P. patens* (PhyscoPII) and from *C. reinhardtii* PII (CrPII) lacking the putative transit signal peptides, starting with amino acid V60 and E63 respectively, with a C-terminally fused Strep-tag II or a N-terminally fused His_6_-tag sequences were overexpressed and purified as described previously^23,24^. The quality of purified PII proteins were checked using SDS-PAGE, and the fractions containing highly purified proteins were combined (Supplementary Fig. S1) and dialyzed in the respective buffer (Supplementary Methods). The proteins concentrations were determined using Bradford reagent (Roti^®^-Quant, Roth). The amino acid sequences of recombinant PII proteins used in this study are described in (Supplementary Fig. S4).

### Expression and purification of ScNAGK, ScPipX and AGPR proteins

The genes encoding NAGK and PipX from *Synechocystis* sp. PCC 6803 and N-acetyl-*γ*-glutamyl-5-phosphate reductase (AGPR) from *E. coli*, were cloned in pET15b plasmid, were induced in *E. coli* strain BL21(DE3), and the N-terminal fused His_6_-tag proteins were purified using affinity chromatography on a Ni-NTA column as described^33,50^.

### Isothermal titration calorimetry (ITC)

The ITC experiments were done using a VP-ITC microcalorimeter (MicroCal, LCC). All details for determination of binding constants for small effector molecules using ITC are fully described in the supplementary information. Heat isotherms of the dilution of the ligand in the cell buffer were collected in a blank run for each experiment in the absence of protein. The binding isotherms were calculated from received data and fitted to one-site and three-sequential binding sites models using the MicroCal ORIGIN software (Northampton) as described^9^. All titrations were performed in duplicates with different purification batches of recombinant PII proteins. The association binding constant (*K*_*a*_) was generated from the software by de-convolution and curve fitting. For calculation of dissociation constant (*K*_*d*_), the *K*_*a*_ value was inversed.

### Coupled NAGK activity assay

The assess the activity of ScNAGK was measured with an enzyme assay, in which the ADP production was coupled to the NADH oxidation by the auxiliary enzymes pyruvate kinase and lactate dehydrogenase according to previously described procedure^32,50^. In a standard assay, 2.4 µg of PpPII protein was added to the reaction mix (50 mM imidazole pH 7.5, 50 mM KCl, 20 mM MgCl_2_, 0.4 mM NADH, 1 mM phosphoenolpyruvate, 10 mM ATP, 0.5 mM DTT, 11 U lactate dehydrogenase, 15 U pyruvate kinase and 50 mM NAG) and the reaction was started by the addition of 6 µg ScNAGK. The oxidation reaction of NADH was recorded for 10 min using spectrophotometer (SPECORD 210 PLUS, Analytik Jena AG) at 340 nm. The oxidation of one molecule of NADH is proportional to the phosphorylation of one molecule of NAG. The NADH molar absorption coefficient is 6178 L mol^−1^ cm^−1^ for at 340 nm, which was used for calculation. The means of triplicates is shown with a standard deviation of less than 5%. From the velocity slopes, the enzymatic constants *K*_*m*_, *K*_*cat*_ and IC_50_ were calculated using the Graph-Pad Prism software (GraphPad Software, USA).

### AGPR-coupled NAGK activity assay

To assay ScNAGK activity in presence of ADP, NAGK-dependent NAG phosphorylation was coupled to the AGPR auxiliary enzyme which catalyze the reduction of NAG-phosphate using NADPH as a cofactor. The change in NADPH absorbance was recorded at 340 nm as described previously^31,33^, in a reaction buffer composed of 50 mM potassium phosphate (pH 7.0), 50 mM KCl, 20 mM MgCl_2_, 0.2 mM NADPH and 0.5 mM DTT. Each reaction contained in a volume of 1 ml 50 mM NAG, 6 *μ*g NAGK and 10 μg of AGPR with/without 2.4 μg of PpPII. The reaction was started by the addition of ScNAGK and was recorded for 10 min with a spectrophotometer (SPECORD 210 PLUS, Analytik Jena). Kinetic constants were calculated as descried above.

### Pull-down analysis

For complex formation, the binding reactions were done in 300 μl of buffer (100 mM Tris HCl, pH 8.0, 150 mM NaCl, 1 mM EDTA) by adding 10 µM of purified proteins: His_6_-ScPipX and Strep-tag ScPII or Strep-tag PpPII. The proteins were mixed in the presence of 1 mM ADP, kept at room temperature for 30 min, and then loaded on StrepTactin spin columns. Unbound proteins were removed by washing ten times per 50 µl by centrifugation with the binding buffer for 30 s, 800 rpm. (MiniSpin, Eppendorf) and bound proteins were eluted in binding buffer contains 10 mM d-desthiobiotin (Sigma-Aldrich). The eluted fractions were analyzed by Tricine-SDS PAGE^51^ and stained with SimplyBlue™ SafeStain (Invitrogen).

### Surface plasmon resonance (SRP) analysis

SPR experiments were conducted using a ProteOn XPR36 system (Bio-Rad) in HBS buffer (10 mM HEPES, 150 mM NaCl, 1 mM MgCl_2_, and 0.005% Tween 20, pH 7.5) at 25 °C. Immobilization of ligand, 1 µM His_6_-PipX (200 µl), was performed on a Ni^+^-loaded HTG sensor chip (Bio-Rad Laboratories) in the vertical orientation into the channel L2, and the continuous running buffer was used at a flow rate of 30 µl/min. The immobilization level was approximately 3000 resonance units (RU). Next, the chip was rotated at 90°, the channels were washed for 3 min with buffer HBS, and 200 nM (200 µl) of strep-tagged PII proteins (ScPII or PpPII) was simultaneously injected in the vertical orientation into different channels at a flow rate of 30 µl/min for an association phase of 400 s, which was followed by a 600 s dissociation phase. Channel L1, which was treated with the buffer without protein, served as a reference. The binding of PII proteins to PipX was recorded as a response signal difference of L2 and L1. All binding sensorgrams were collected, processed and analyzed using the integrated ProteOn Manager software (Bio-Rad Laboratories).

## Electronic supplementary material

Supplementary information
