# Supplementary material for: The PII signaling protein from red algae represents an evolutionary link between cyanobacterial and Chloroplastida PII proteins
Source: Sci Rep. 2018 Jan 15;8:790. doi: 10.1038/s41598-017-19046-7 (PMC5768801; doi:10.1038/s41598-017-19046-7)
Supplement: Supplementary file 1 — Supplementary information [file 41598_2017_19046_MOESM1_ESM.pdf]

Supplementary Information

The PII signaling protein from red algae represents an evolutionary link between cyanobacterial and Chloroplastida PII proteins

Tatyana Lapina[1], Khaled A. Selim[2], Karl Forchhammer[2], Elena Ermilova[1]

Supplementary Methods

Determination of Binding Constants for Small Effector Molecules using Isothermal Titration Calorimetry

The binding of the small effector molecules to the recombinant PII proteins was tested using isothermal titration calarmity (ITC); VP-ITC microcalorimeter (MicroCal). All titrations were performed on 20°C using 0.298 µl injection syringe with injection 6 µl or 10 µl of the tested ligands for 45 or 29 times, respectively into the cell (1.4 ml) with continuous stirring at 155 rpm. All tested ligands were high grade, prepared freshly in the same buffer of the protein and purchased from Sigma-Aldrich. Heat isotherms for the dilution of the ligand in the cell buffer were collected in a blank run in the absence of protein. The received calorimetric data were analyzed using MicroCal Origin software and fitted into one-site and three-sequential binding sites models, as indicated by the manufacture for calculation of binding isotherms[9]. For data reproducibility, the titrations were performed in duplicates with different purification batches of recombinant PII proteins. The association binding constant (K_a) was generated from the software by de-convolution and curve fitting. For calculation of dissociation constant (K_d), the K_a value was inversed.

For determination the binding isotherms of red algal PpPII protein, a buffer system composed of 20 mM Tris-HCl, 150 mM NaCl, 5 mM $MgCl_2$, 0.5 mM EDTA, and 10% glycerol (pH 7.7) was used. For determination ATP and ADP binding isotherms upon the binding to PpPII protein, His-tagged version of recombinant PpPII protein (≈ 28.3 µM trimeric concentration) was titrated against 2 mM ATP or 2 mM ADP. For determination of ATP and ADP binding isotherms in presence of 1 mM 2-OG, His-tagged recombinant PpPII protein (20 or 9 µM trimeric concentration) was titrated against 0.5 mM of ATP or 1 mM ADP, respectively. However, for determination of 2-OG binding isotherm, strep-tagged version of recombinant PpPII protein solution (23.3 µM trimeric concentration) was titrated against 1 mM solution of 2-OG in presence of 1 mM of ATP or 1 mM ADP.

For determination the binding isotherms of cyanobacterial SyPII protein, a strep-tagged recombinant protein (20 µM trimeric concentration) was overexpressed and used as described previously[9, 15], in a buffer composed of 10 mM Hepes-NaOH, 50 mM KCl, 50 mM NaCl, and 1 mM $MgCl_2$ (pH 7.4). For determination ATP and 2-OG binding isotherms, the recombinant SyPII protein was titrated against 1 mM ATP or 1 mM 2-OG (in presence of 2 mM ATP). For determination of ATP and ADP binding isotherms in presence of 1 mM 2-OG, the SyPII protein was titrated against 0.5 mM ATP or 1 mM ADP. For determination, the threshold level of ADP that can relieve the 2-OG inhibitory effect on ADP, SyPII protein was titrated again against 1 mM ADP (in presence of 1 mM 2-OG and 150 µM ADP).

For determination the binding isotherms of green algal CrPII protein, a His- and strep-tagged versions of recombinant protein was used in system composed of 20 mM Tris-HCl, 150 mM NaCl,

5 mM $MgCl_2$, 0.5 mM EDTA, and 10% glycerol (pH 7.6). For determination ATP and ADP binding isotherms, the strep-tagged recombinant CrPII protein was titrated against 2 mM ATP or 2 mM ADP. For determination of ATP and ADP binding isotherms in presence of 2 mM 2-OG, both of His- and strep-tagged versions of CrPII (45 µM trimeric concentration) was titrated against 1 mM of ATP or 1 mM ADP. For determination of 2-OG binding isotherm, the recombinant CrPII protein solution was titrated against 1 mM 2-OG in presence of 2 mM ATP or 2 mM ADP.

For determination the binding isotherms of plant-moss PhyscoPII protein, a strep-tagged recombinant protein (5 or 6 µM trimeric concentration) was used in system composed of 20 mM Tris-HCl, 150 mM NaCl, 5 mM $MgCl_2$, and 0.5 mM EDTA (pH 8.0). For determination ATP and ADP binding isotherms, the recombinant PhyscoPII protein was titrated against 1 mM ATP or 1 mM ADP. For determination of ATP and ADP binding isotherms in presence of 2-OG, PhyscoPII was titrated against 1 mM of ATP or 1 mM ADP. Additionally, 1 mM 2-OG was titrated in presence of 1 mM ATP.

<u>**Supplementary Figures**</u>

Supplementary Figure S1

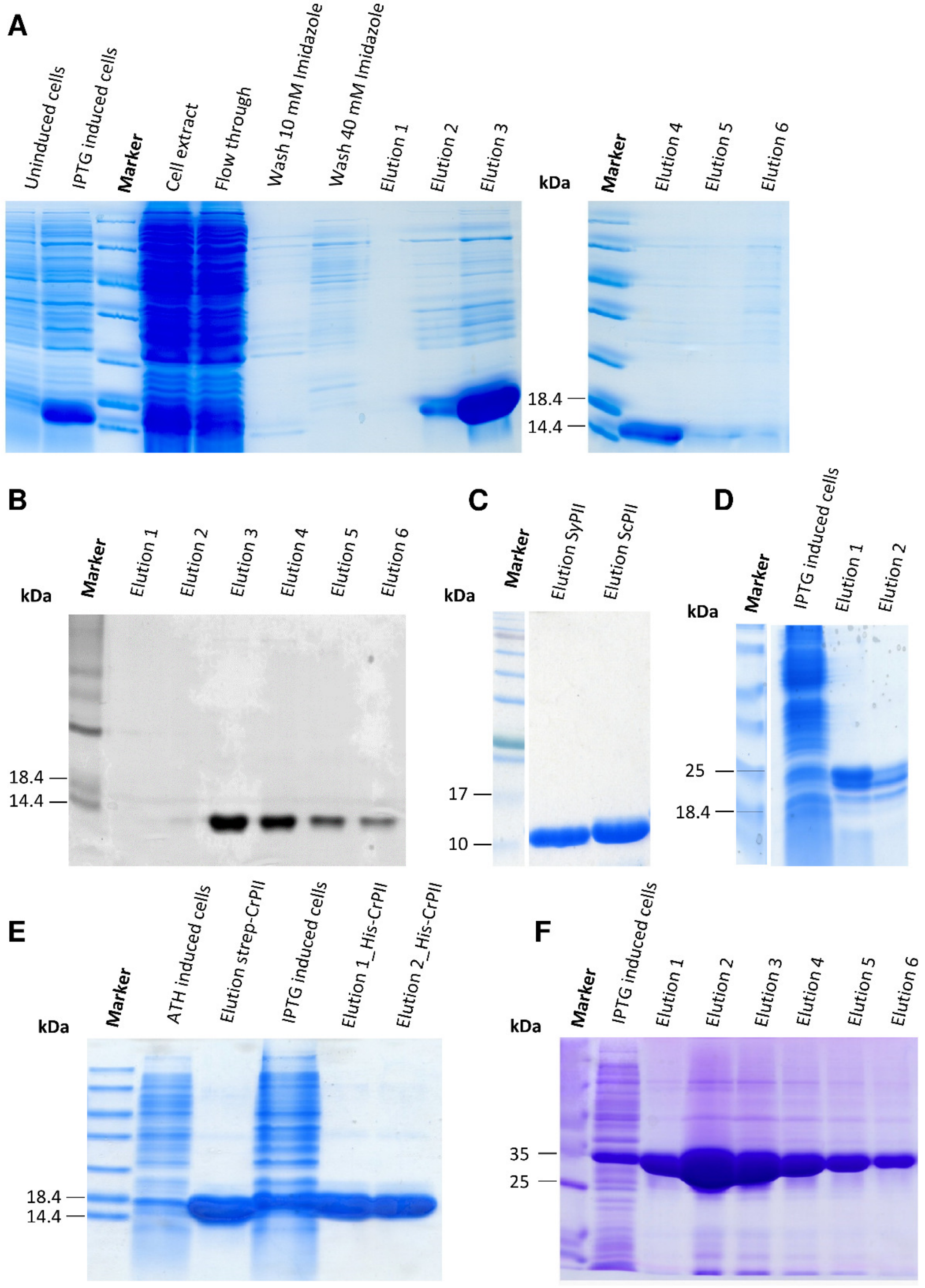

Figure S1. SDS-PAGE of purified PII proteins and NAGK. (A) Purification of recombinant His-tagged PpPII protein (elution fractions 2-4 combined together). (B) Purified recombinant

Strep-tagged PpPII protein (elution fractions 3-6 combined together). (C) Purified recombinant cyanobacterial SyPII and ScPII proteins. (D) Purification of recombinant plant PhyscoPII protein (elution fractions 1 and 2 combined together). (E) Purification of green algal Strep- and His-tagged CrPII protein (elution fractions 1 and 2 of His-tagged CrPII combined together). (F) Purification of ScNAGK. The molecular weight of monomeric His-tagged PpPII, Strep-tagged PpPII, Strep-tagged SyPII, Strep-tagged ScPII, Strep-tagged PhyscoPII, Strep-tagged CrPII, His-tagged CrPII, and ScNAGK proteins are 14.3, 13.5, 13.6, 13.6, 20.2, 17.0, 17.9, and 33.6 kDa, respectively.

Supplementary Figure S2

```
Physcomitrella   1 MASWKSVSTVLAKEPIGIRSAPAELIKG---TSSVKFSYPAKKARFQRARVEAVTDQTARQAAVRTSQYTG
Arabidopsis      1 MATVT-----SNASPKSFSFTVSNPFKTLIPNKSPSLCYPTRNKNHHRLGFSIKATVSTPPSIATGNAPSP
Chlamydomonas    1 -----------MALLAAKTTSPSVTTRRSVTGLPSVSGFRASRPTRKHGIYVRAMAAATAEEAELRKQLTN
Chlorella        1 -----------MAMVPCPSQRLSLSSSIKSAAPHQICVSRPSRAPRRPAHLAVTAAAAAPQASKALDRFSA
Synechococcus    1 -------------------------------------------------------------MSSEFIEAGA
Synechocystis    1 -----------------------------------------------------------MSSTQDYIGEEA
Porphyra         1 -------------------------------------------------------------------MLTN
Pyropia          1 -------------------------------------------------------------------MLTN
Galdieria        1 ------------------------------------------MIIRINFIKYKKQNNMNNNHFNIDQSVNN
Gracilaria       1 -------------------------------------------------------------------MLNN

Physcomitrella  69 KERVDILAEALPFIQRFQGKTVVVKYGGAAMKDESLKDGVIKDLVLLSCVGMKCVLVHGGGPEINQWLGKL
Arabidopsis     67 DYRVEILSESLPFIQKFRGKTIVVKYGGAAMTSPELKSSVVSDLVLLACVGLRPILVHGGGPDINRYLKQL
Chlamydomonas   61 LDRVTILSEALPYLQKFRGKTVVVKYGGAAMKDPTLKAGVISDLVLLSCVGIRCVLVHGGGPEINSWLAKV
Chlorella       61 FDRVSVLSEALPYLQRFRGKTIVIKYGGAAMKDESLKARVVSDLVLLSCVGIHPVMVHGGGPEINSWLNKL
Synechococcus   11 ADRVRILSEALPYLQQFAGRTVVVKYGGAAMKQEELKEAVMRDIVFLACVGMRPVVVHGGGPEINAWLGRV
Synechocystis   13 ATRVKILSEALPYIQHFAGRTVVVKYGGAAMKDSNLKDKVIRDIVFMASVGIRPVVVHGGGPEINTWLDKV
Porphyra         5 TERVKVLSDVT-ILQKFSSRIIVIKYGGAAMKNQKLKDHVISDLVFLSFIGLRPILVHGGGPEINFWLDQL
Pyropia          5 SERVKVLSEALPYIQQFSSRIIVIKYGGAAMKNQKLKDQVISDLVFLSFIGLRPILVHGGGPEINFWLDRL
Galdieria       30 LNRVQILSEALPYIQKFAGSTIVIKYGGSIMTNNKLKESIVNDIILLSHVGIKPILIHGGGPEINSWLEKL
Gracilaria       5 FERVQILSDALPFVQRFHGRTLVVKYGGSAMKNVYLKSKIIEDILFLSYIGIKVVIVHGGGPMINHWLKQV

Physcomitrella 140 GIEPHFKNGLRVTDAATMEVVEMVLVGKVNKSLVSLINKAGGCAVGMCGKDGKLIKARPLS--DDLGFVGE
Arabidopsis    138 NIPAEFRDGLRVTDATTMEIVSMVLVGKVNKNLVSLINAAGATAVGLSGHDGRLLTARPVPNSAQLGFVGE
Chlamydomonas  132 GIEAVFKNGLRVTDAATMEIVEMVLGGRVNKSLVSLIQQAGGKAVGLTGKDGQLLRARQMV-ELDIGYVGE
Chlorella      132 GIEAQFKNGLRVTDAATMDVVEMVLGGRVNKSLVSLIQQSGGAAVGLCGKDSDIIRARQMV-EKDIGFVGE
Synechococcus   82 GIEPQFHNGLRVTDADTMEVVEMVLVGRVNKDIVSRINTTGGRAVGFCGTDGRLVLARPHD-QEGIGFVGE
Synechocystis   84 GIEPQFKDGLRVTDAATMDIVEMVLVGRVNKELVNLINQAGGKAVGLCGKDGQLMTARTMT-NKDVGFVGE
Porphyra        75 KILPKFENGVRVTDQPTMDIVEMVLVGRVNKDLVASINKQGGKSVGLSGKDGLLITSRPSD-KPNLGFVGE
Pyropia         76 KILPKFDDGVRVTDQPTMDIVEMVLVGRVNKDLVATINKQGGKSVGLSGKDGLLITPRPNG-KANLGFVGE
Galdieria      101 NIKSKFKNGIRITDSNTIEIVEMVLAGKINKEIVSLINKQGSYAVGLTGKDGKLIQCESEE-DIEVGLVGK
Gracilaria      76 NIQPKFFNGIRVTDKDTMELVEMVLVGKVNKDLVTLLNRSSNLAVGLSGKDANLVVASSFFPDQKDNYTGK

Physcomitrella 209 ITAVDTSVVKGILNSGHLPVIATVAADNDGQAYNINADTAAGEIAASLGAEKLILMTDVQGLMLDHKDSST
Arabidopsis    209 VARVDPSVLRPLVDYGYIPVIASVAADDSGQAYNINADTVAGELAAALGAEKLILLTDVAGILENKEDPSS
Chlamydomonas  202 VTKVDPTILNVLVEDEYIPVVATIATDYSGQALNINADTAAGEIAAALKAEKLVLMTDVPGVLRDKNDIGT
Chlorella      202 VTSVNPSLLQTLVADGYIPVVASVASDGKGQGLNVNADTAAGEIAASLRAEKLILMTDVPGVMRDKDDPST
Synechococcus  152 VNSVNSEVIEPLLERGYIPVISSVAADENGQSFNINADTVAGEIAAALNAEKLILLTDTRGILEDPKRPES
Synechocystis  154 VSSVDARVVETLVKSGYIPVISSVAADEFGQAHNINADTCAGELAAALGAEKLILLTDTRGILRDYKDPST
Porphyra       145 VQNVDTNLLEILINNNYIPVIASVAADKQGQSYNINADTVAGEIAARLNAEKLILLTDTPGILRNASDATT
Pyropia        146 VQNIDTKLLKILINNNYIPVIASVAADKEGQSYNINADTVAGEIAAALNAEKLILLTDTPGILRNSSDPST
Galdieria      172 IKHINTNIIKTIINAGYIPVIASIGSDLDGKSYNINADTVAGEIAVSINAEKLILLTNTAGILNDINNNDS
Gracilaria     147 VQQVNIEIVNLLLSSGYIPVVASVASDLNGQAYNINADSVAGAIAECLNAEKLILLTDTPGIMSDINDPSS

Physcomitrella 280 LVPEVNIKGVRKLIEDGIVTGGMIPKVECCVKSLAQGVHSTHIIDGRAPHSLLLEILTDEGAGTMITG---
Arabidopsis    280 LIKEIDIKGVKKMIEDGKVAGGMIPKVKCCIRSLAQGVKTASIIDGRRQHSLLHEIMSDEGAGTMITG---
Chlamydomonas  273 KIQALDIRSCRELIQDGVIAGGMIPKIECCIRCLSQGVKAAHIIDGRASHSILMELLTDEGVGTMITG---
Chlorella      273 KFAALTIRECKELEDQGIIAGGMIPKVDCCIRSLSQGVSATHIIDGRQPHSLLMELLTDEGIGTMICERPL
Synechococcus  223 LIPRLNIPQSRELIAQGIVGGGMIPKVDCCIRSLAQGVRAAHIIDGRIPHALLLEIFTDAGIGTMIVGSGY
Synechocystis  225 LIHKLDIQQARELIGSGIVAGGMIPKVTCCVRSLAQGVRAAHILDGRLPHALLLEVFTDLGIGSMIVASGY
Porphyra       216 LISHLSIQEARDLTKTAVISGGMIPKVNCCIRSLAQGVASAHILDGRIDHALLLEILTDQGIGSMLVV---
Pyropia        217 LISHLNIQEARDLTQTAVISGGMIPKVNCCIRSLAQGVASAHILDGRIDHALLLEILTDQGIGSMLVV---
Galdieria      242 LIRLLNIEDIKSLITKRKISGGMIPKVNCCMRALAKGLPAAHIIDGRVFHALLLEIFTDQGIGSMLVNSSN
Gracilaria     218 LIKYLNISQLEELKSQKIILGGMIPKVDCCIKALQGNVSSAHIIDGSVEHALLLEILTSAGIGSMLVL---

Physcomitrella 351 --------
Arabidopsis    351 --------
Chlamydomonas  344 --------
Chlorella      344 --------
Synechococcus  294 HEAHQPWQ
Synechocystis  296 DL------
Porphyra       287 --------
Pyropia        288 --------
Galdieria      313 QYINSKH-
Gracilaria     289 --------
```

Figure S2. Multiple sequence alignment of the deduced amino acids of NAGK homologs from selected plants: *Physcomitrella patens* and *Arabidopsis thaliana*, green algae: *Chlamydomonas*

reinhardtii and *Chlorella variabilis*, cyanobacteria: *Synechococcus elongatus* PCC 7942 and *Synechocystis* sp. PCC 6803, red alga containing PII: *Porphyra purpurea* and *Pyropia yezoensis*, and red alga lacking PII: *Galdieria sulphuraria* and *Gracilaria enuistipitata.* Residues highlighted in black are identical or highly conserved in all aligned NAGK proteins. Amino acids in a gray background represent similar residues. The alignments revealed absence of putative N-terminal transit peptide sequences with more than 62% sequence identity within cyanobacterial and red algal NAGK. The Arg, the key residue for initiation PII-NAGK complex formation, is highlighted in yellow and indicated in red, and it is not conserved among red algal lacking PII. The alignment was made using Clustal Omega program and refined manually.

Supplementary Figure S3

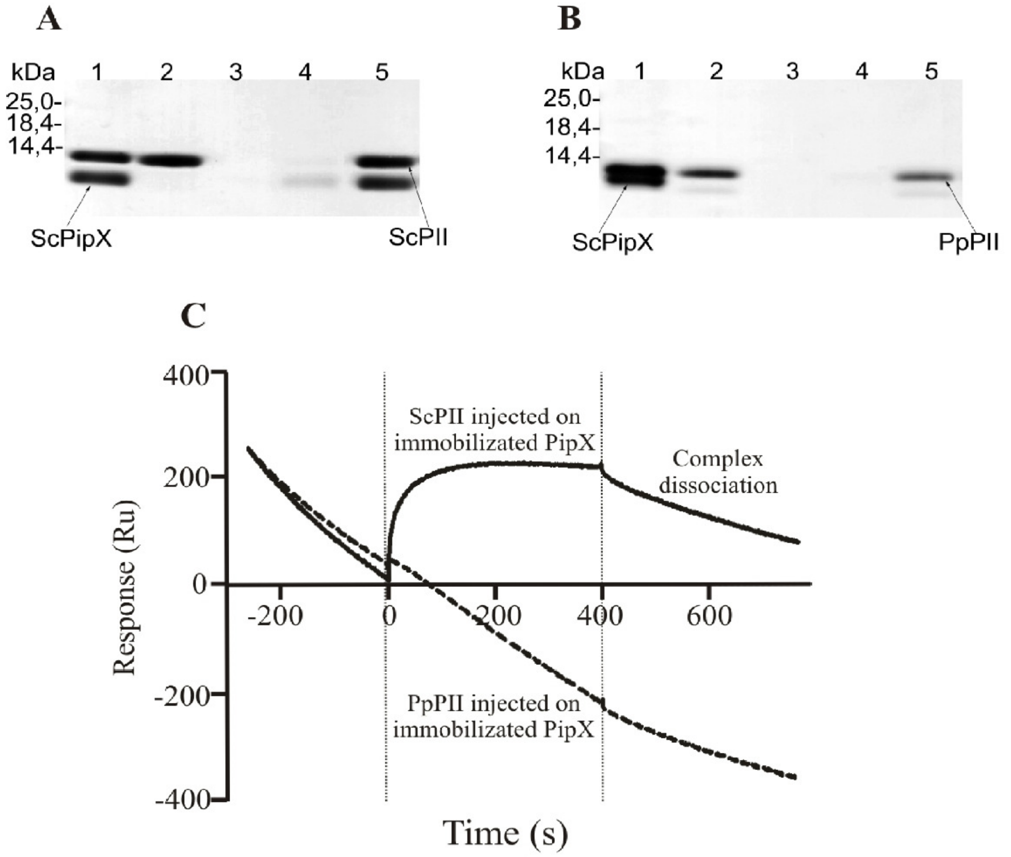

Figure S3. Analysis of PipX binding to PII proteins. Complex formation between PipX and ScPII (A) or PpPII (B) was assessed by pull-down. Reactions were performed in the presence of 1 mM ADP. The proteins were eluted with desthiobiotin and the eluted fractions were subjected to SDS-PAGE, the gel was stained using SimplyBlue™. Lane 1, control mixture PipX with ScPII or PpPII before chromatography; lane 2, ScPII or PpPII; lane 3, negative control His_6-PipX loaded on StrepTactin spin column; lane 4, the last washing fraction; lane 5, complex elution ScPII or PpPII plus PipX. (C) Complex formation between PipX and PII proteins was assessed by SPR. ScPipX was bound on a Ni-HTG sensor chip in the vertical orientation (see Materials and methods) to give an increase in RU of approximately 3000 RU. ScPipX was pre-incubated 1 mM ADP. ScPII (solid line) and PpPII (dashed line) proteins were injected as analytes in the horizontal orientation. Channel L2 was used as background control.

Supplementary Figure S4

A

MGSSHHHHHHSSGLVPRGSMKKIEAIIRPFKLNEVKLALVKGGIGGMTVVKVSGFGRQKGQTERYKGSEYSIDIIDKIKIEII
VSDDKVNSITEIIIKTAKTGEIGDGKIFISDVEQVIRIRTNDLNSAAL*

B

MKKIEAIIRPFKLNEVKLALVKGGIGGMTVVKVSGFGRQKGQTERYKGSEYSIDIIDKIKIEIIVSDDKVNSITEIIIKTAKTGEIG
DGKIFISDVEQVIRIRTNDLNSAALSAWSHPQFEK*

C

MKKIEAIIRPFKLDEVKIALVNAGIVGMTVSEVRGFGRQKGQTERYRGSEYTVEFLQKLKLEIVVEDAQVDTVIDKIVAAART
GEIGDGKIFVSPVDQTIRIRTGEKNADAISAWSHPQFEK*

D

MKKVEAIIRPFKLDEVKIALVNAGIVGMTVSEVRGFGRQKGQTERYRGSEYTVEFLQKLKIEIVVDEGQVDMVVDKLVSAA
RTGEIGDGKIFISPVDSVVRIRTGEKDTEAISAWSHPQFEK*

E

MELESIQCDLSAFPGVKFFRIEAIFRPWRLPFVIDTLSKYGIRGLTNTPVKGVGVQGGSRERYAGTEFGPSNLVDKEKLDIV
VSRAQVDAVVRLVAASAYTGEIGDGKIFVHPVAEVVRIRTAETGLEAEKMEGGMEDMMKKKKSAWSHPQFEK*

F

MGSSHHHHHHSSGLVPRGSMELESIQCDLSAFPGVKFFRIEAIFRPWRLPFVIDTLSKYGIRGLTNTPVKGVGVQGGSRE
RYAGTEFGPSNLVDKEKLDIVVSRAQVDAVVRLVAASAYTGEIGDGKIFVHPVAEVVRIRTAETGLEAEKMEGGMEDMMK
KKK*

G

MVASASDPKSPNWRKRVSGVVQVHLEEYDFDDQSKDYQPSVDFYKVEAVLRPWRLSPVSSALLKMGIRGVTVTDVRGF
GAQGGSRERQAGTEYAGDSYLKKVKLEIVVSKDQVEAVIDTIIDQARTGEIGDGKIFVSPVSDIIRIRTGERGLKAERMAGG
RAAMQTSAEGSDGNSAWSHPQFEK*

H

MGSSHHHHHHSSGLVPRGSMSSTQDYIGEEAATRVKILSEALPYIQHFAGRTVVVKYGGAAMKDSNLKDKVIRDIVFMAS
VGIRPVVVHGGGPEINTWLDKVGIEPQFKDGLRVTDAATMDIVEMVLVGRVNKELVNLINQAGGKAVGLCGKDGQLMTA
RTMTNKDVGFVGEVSSVDARVVETLVKSGYIPVISSVAADEFGQAHNINADTCAGELAAALGAEKLILLTDTRGILRDYKD
PSTLIHKLDIQQARELIGSGIVAGGMIPKVTCCVRSLAQGVRAAHILDGRLPHALLLEVFTDLGIGSMIVASGYDL*

Figure S4. Amino acid sequences of the proteins used in this study. (A) His-tagged PpPII protein. (B) Strep-tagged PpPII protein. (C) Strep-tagged SyPII protein. (D) Strep-tagged ScPII

protein. (E) Truncated version of Strep-tagged CrPII protein lacking the transit signal peptides and starting from E63. (F) Truncated version of His-tagged CrPII protein lacking the transit signal peptides and starting from E63. (G) Truncated version of Strep-tagged PhyscoPII protein lacking the transit signal peptides and starting from V60. (H) His-tagged ScNAGK. The tag sequences are highlighted in yellow.
